# One Year Longitudinal Assessment of Subjective and Objective Accommodation After Phakic IOL Implantation

**DOI:** 10.3390/vision10020022

**Published:** 2026-04-16

**Authors:** Esther López-Artero, María García-Montero, Blanca Poyales, Ricardo Pérez-Izquierdo, Alba Sáez, Nuria Garzón

**Affiliations:** 1 Miranza Group, C/Galileo 104, 28003 Madrid, Spain; marilo69@ucm.es (E.L.-A.); blanca.poyales@miranza.es (B.P.); ricardo.perez@miranza.es (R.P.-I.); alba.saez@miranza.es (A.S.); 2 Optometry and Vision Department, Faculty of Optics and Optometry, Complutense University of Madrid, 28037 Madrid, Spain; mgarzonj@ucm.es

**Keywords:** phakic intraocular lens, implantable collamer lens, accommodation, optical quality, myopia

## Abstract

Purpose: To evaluate the 1 year behavior of accommodation and optical quality one year after the implantation of phakic intraocular lenses, specifically the implantable collamer lens (ICL), in myopic patients, comparing outcomes between low- and high-myopia groups. Methods: This comparative longitudinal study included 38 eyes of 38 patients who underwent ICL implantation for myopia correction. Patients were divided into two groups based on preoperative manifest sphere: low myopia (−2.50 D to −6.25 D) and high myopia (>−6.25 D to −12.50 D). The amplitude of accommodation (AA), subjective accommodative response (AR), optical quality parameters including the modulation transfer function (MTF) cut-off, objective scatter index (OSI) and Strehl ratio (SR), and objective accommodative response with a double-pass system (HD Analyzer, Visiometrics) were assessed preoperatively, 1 month, and 1 year postoperatively. Results: Both groups achieved postoperative refractive outcomes close to emmetropia, with high efficacy and safety indices. A statistically significant decrease in the amplitude of accommodation was observed at 1 month and remained stable at 1 year in both groups; however, this change was not clinically meaningful. The optical quality parameters (MTF cut-off, OSI, and Strehl ratio) and objective accommodative response with the HD Analyzer showed no clinically relevant changes over time, with no significant intergroup differences detected (*p*-value > 0.05). Conclusions: An initial reduction in accommodative amplitude was observed after ICL implantation without recovery over time; however, it was not clinically relevant, as it fell within the test–retest variability in the minus lens technique. Other accommodative parameters and optical quality remained stable at 1 year in both low and high myopia.

## 1. Introduction

Refractive surgery for low and high myopia correction has evolved significantly over the past few decades. Phakic intraocular lenses (p-IOLs) have become a safe and effective surgical alternative for patients who are not eligible for corneal refractive procedures. Among these, the posterior chamber p-IOL, the implantable collamer lens (ICL; STAAR Surgical Inc, Monrovia, CA, USA), offers excellent visual performance and is particularly valued for its high optical quality, reversibility, and preservation of corneal integrity [1,2,3,4].

Beyond visual and refractive outcomes, there has been growing clinical interest in understanding how ICL implantation impacts other visual functions, particularly accommodation and optical quality [5,6]. This is especially relevant for postoperative young adult patients, who, despite retaining the capacity for active accommodation, may experience visual fatigue or increased effort when working at near distances [7,8].

Previous studies have reported that accommodation is maintained or minimally impaired in the short term after ICL implantation. The small reduction may be due to either the increased accommodative effort following the change in refractive state, or to the transient dysfunction of the ciliary muscles after ICL fixation [9,10]. However, evidence remains limited regarding the 1 year behavior of accommodation following ICL implantation and whether this behavior varies according to the degree of preoperative myopia.

Considering that high myopes typically have a larger ciliary muscle ring diameter and different ocular biomechanics [11], and that corrected myopic eyes tend to have a higher accommodative lag than emmetropic or hyperopic eyes corrected with spectacles or contact lenses [12], it is plausible that their accommodative response may differ between myopia groups.

Additionally, optical quality after surgery is not only a function of corrected visual acuity, but also involves factors such as intraocular scattering and modulation transfer function (MTF), which may influence patient satisfaction and near vision performance and accommodation [13]. Devices such as the HD Analyzer (Visiometrics SL, Terrassa, Spain) allow objective quantification of these parameters, offering a more complete understanding of postoperative optical quality. This can assist in the objective evaluation of the medium-term accommodative response following myopia correction through ICL implantation [14].

Therefore, this study aimed to evaluate and compare 1 year changes in accommodative function, as assessed by objective and subjective tests and optical quality, following p-IOL EVO Visian ICL implantation in patients with low and high myopia.

## 2. Materials and Methods

### 2.1. Patients

This prospective longitudinal study was performed at Miranza IOA Clinic (Madrid, Spain) from March 2022 to May 2024. A total of 38 myopic patients who underwent spherical p-IOL EVO Visian ICL and had completed regular visits over 12 months were included in this study. This study was approved by the local Ethics Committee of the Hospital Clínico San Carlos, Madrid, Spain (CI. 22/236-O_P_Tesis), and adhered to the tenets of the Declaration of Helsinki. All patients participating read and signed an informed consent form.

Inclusion criteria were myopia from −2.50 to −18.00 diopters (D) and regular astigmatism lower than 1.25 D, age from 20 to 40 years, and preoperative corrected distance visual acuity (CDVA) better than 0.1 logMAR. Exclusion criteria included anterior chamber depth (ACD) < 2.8 mm, endothelial cell density (ECD) < 2.000 cells/mm^2^, manifest strabismus, accommodation or binocular disorders, cataract, history of glaucoma, previous intraocular surgery, altered or unstable tear film or other ophthalmic diseases, and systemic pathology that could affect the accommodative system or pharmacological treatments with side effects on accommodation. Patients were divided into two groups based on the preoperative manifest sphere of myopia: low-myopia group (from −2.50 D to −6.25 D) and high-myopia group (>−6.25 D) [15].

The sample size was calculated based on the amplitude of accommodation (AA) 1 year after p-IOL ICL implantation, assuming a two-tailed test with a standard deviation of 2.85 D, according to the published results of Kamiya et al. [16] relating to the AA 1 year postoperatively. Accepting an alpha risk of 0.05, a statistical power greater than 0.8 and a drop-out rate of 5%, a total of 19 subjects per group were required to detect a statistically significant difference of 2.00 D or greater.

### 2.2. ICL Selection and Surgical Procedure

The EVO Visian ICL is a posterior chamber p-IOL designed for implantation between the posterior surface of the iris and the anterior capsule of the crystalline lens, with its haptics positioned in the ciliary sulcus. Composed of a proprietary biocompatible material known as collamer, it features a central port measuring 0.36 mm in diameter. This facilitates aqueous humor flow and eliminates the need for peripheral iridotomies.

This lens model is available in four diameters: 12.1 mm, 12.6 mm, 13.2 mm and 13.7 mm, providing spherical corrections for myopia ranging from –0.50 D to –18.00 D at the corneal plane.

Lens sizing and power calculations were performed using STAAR Surgical’s online platform (https://ocos.staarag.ch/landing/, accessed on 2 February 2025) to target emmetropia in all cases. The calculation protocol incorporated parameters such as manifest refraction, anterior chamber depth, keratometry readings, white-to-white distance and central corneal thickness (pachymetry), to optimize lens selection and postoperative outcomes.

The pupil was dilated 30 min before the surgical procedure. Under topical anesthesia, a superior or inferior corneal paracentesis incision was made and the ICL was implanted in the posterior chamber through a 2.8 mm temporal corneal incision. The viscoelastic surgical material was then completely washed out using balanced salt solution and a miotic agent (acetylcholine) was instilled. Postoperative medications included topical antibiotics and steroids. All procedures were performed by the same expert surgeon (BP) and were uneventful and free of complications.

### 2.3. Preoperative and Postoperative Visual Assessment

All patients underwent a comprehensive preoperative ophthalmological assessment before surgery. This protocol included a detailed clinical history, manifest and pharmacological refraction under tropicamide 1% mydriasis (performed 30 min after the instillation of three drops of tropicamide 1% administered at 5 min intervals), slit lamp biomicroscopy, and intraocular pressure (IOP) measurement. Additionally, central endothelial cell density was assessed using the CEM-530 specular microscope (Nidek Co., Ltd. Gamagori, Aichi, Japan), and corneal topography, mesopic pupil diameter, anterior chamber depth and width were evaluated using the Pentacam HR system (Oculus Optikgeräte GmbH, Wetzlar, Germany). A thorough dilated fundus examination was also performed in all cases.

Uncorrected distance visual acuity (UDVA) and corrected distance visual acuity (CDVA), both measured with a Snellen chart and expressed in LogMAR, as well as manifest refraction, spherical equivalent (SE) refractive error, subjective accommodation measurements, optical quality parameters and objective accommodative response, were assessed preoperatively—at a separate visit without pharmacological interference, at 1 month and 1 year postoperatively. Intraocular pressure (IOP) and anterior segment status were evaluated at each follow-up visit.

Postoperative dynamic changes in the vault following implantation were assessed using anterior segment optical coherence tomography (OCT) (CASIA2, Tomey Corporation, Nagoya, Japan). Vault measurements were obtained with the OCT scan beam centrally aligned to minimize both extrinsic and intrinsic distortions, thereby preventing aspect ratio-related measurement bias. The vault interval was defined as the range between the central vault measurements obtained under conditions of maximal mydriasis and maximal miosis induced by controlled light stimulation. The vault range was defined as the absolute difference between these two measurements.

### 2.4. Subjective Accommodation Assessment

The monocular amplitude of accommodation was assessed using the minus lens technique. This test was performed at a distance of 33 cm using the Early Treatment Diabetic Retinopathy Study (ETDRS) chart 2000 (Precision Vision, Inc., Purvis, MS, USA) under uniform lighting conditions (ambient luminance ≥ 120 cd/m^2^, Weber contrast ≥ 90%, uniformity Lmin/Lmax ≥ 80%). Participants were asked to read two lines larger (i.e., lower visual acuity demand) than their distance-corrected near visual acuity (DCNVA). Minus spherical lenses were then added incrementally in steps of 0.25 D until the first sustained blur was reported. The amplitude of accommodation was calculated by combining the total dioptric power of the added minus lenses with 2.50 D, to account for the accommodative demand at 33 cm and to compensate for the minification effects induced by the negative lenses [17].

Subjective accommodative response, specifically the accommodative lag, was evaluated using the fused cross-cylinder (FCC) method based on a previous study [18]. Participants viewed a cross-grid target at a working distance of 40 cm under mesopic lighting and binocular conditions. A ±0.50 D fused cross-cylinder was positioned in front of both eyes, with its negative axis oriented at 90° under photopic conditions. Depending on whether the participant could see the horizontal or vertical lines more clearly, plus or minus spherical lenses were introduced in 0.25 D increments until the lines appeared equally distinct. Clearer perception of the horizontal lines indicated the presence of accommodative lag.

### 2.5. Optical Quality Assessment—HD Analyzer

The optical quality parameters were measured using the High-Definition (HD) Analyzer double-pass retinal imaging system. This technology can objectively assess optical quality metrics by capturing the retinal point spread function (PSF), which is generated by a monochromatic point source stimulus after reflection and double passage through the ocular media [19]. The device incorporates an internal Badal optical system that controls vergence precisely across the tested range, ensuring a standardized stimulus regardless of residual accommodative fluctuations. Additionally, all measurements were performed under mesopic conditions (3.0–3.5 lux) with a fixed 4.0 mm artificial pupil, which minimizes the contribution of tonic accommodation and reduces inter-subject variability. The parameters evaluated included the objective scatter index (OSI), MTF cut-off frequency and the Strehl ratio (SR), representing the ratio of the measured PSF peak intensity to that of an aberration-free system. Examinations were performed under non-cycloplegic conditions to preserve the physiological accommodative state of the eyes during testing and using the patient’s manifest refraction.

### 2.6. Objective Accommodative Response

The objective assessment of accommodative response was performed using the HD Analyzer which conducted a dynamic vergence sweep ranging from +1.00 diopter (D) to –3.00 D, relative to the subject’s manifest refraction. The device sequentially acquires point spread function (PSF) images of the retina at 0.50 D intervals across this range. At each vergence step, the system captures a double-pass image and calculates the width of the profile (WP) distribution at 50% of its maximum value, expressed in minutes of arc. This parameter correlates inversely with optical quality: higher values indicate lower quality. An internal Badal system precisely controls the vergence shift to maintain the stimulus’s constant angular size during changes in accommodation. The corresponding optical quality is recorded for each accommodative demand and used to generate a normalized image quality profile. All measurements were taken monocularly under mesopic conditions with a 4 mm artificial pupil diameter. Each defocus step was analyzed independently as a repeated measure; no summary slope or composite index was derived across steps.

The lighting conditions for all tests were controlled and verified by an external photometer (Gossen Starlite II, Foto- und Lichtmesstechnik GmbH, Nuremberg, Germany), ensuring adequate luminance, contrast and uniformity consistent with requirements.

### 2.7. Statistical Analysis

Statistical analyses were conducted using SPSS software version 22.0 (IBM Corp., Armonk, NY, USA). Although measurements were obtained from both eyes, only data from the right eye were included for analysis to maintain statistical independence [20]. Descriptive statistics were presented as mean ± standard deviation and range calculation. Data distribution for all quantitative variables was assessed using the Shapiro–Wilk test to determine normality. Pairwise comparisons were performed to assess the effect of treatment over time. Specifically, three pairwise comparisons were conducted: baseline vs. 1 month, baseline vs. 1 year, and 1 month vs. 1 year.

Depending on data distribution, paired *t*-tests or Wilcoxon signed-rank tests were applied. Bonferroni correction was used to adjust for multiple testing. Bonferroni correction was applied across the three pairwise time point comparisons, yielding an adjusted threshold of α = 0.017. For independent group comparison, the unpaired *t*-test or the Mann–Whitney U test was applied, depending on data normality. The Pearson correlation test was used to analyze the relationship between the ICL vault and AA change. All reported *p*-values correspond to bilateral (two-tailed) statistical tests unless otherwise specified. The threshold for statistical significance was set at *p*-value < 0.05.

## 3. Results

The study enrolled 38 patients in the total group, and the mean age was 31.97 ± 5.36 years (range 20 to 40 years). There were no significant differences in age between groups (*p* = 0.146). The mean preoperative SE was −5.15 ± 1.14 D (range −2.88 D to −6.62 D) in the low-myopia group, and −8.55 ± 2.10 D (range −6.50 D to −12.25 D) in the high-myopia group. In both groups, the mean calculated refractive SE demonstrated the smallest deviation from zero (emmetropia), being 0.05 ± 0.17 D in the low-myopia group and 0.06 ± 0.18 D in the high-myopia group.

Demographic data are summarized in Table 1. The mean residual refractive error SE was 0.19 ± 0.20 D and 0.18 ± 0.18 D in the low-myopia group, and 0.18 ± 0.23 D and 0.11 ± 0.22 D in the high-myopia group at 1 month and 1 year after surgery, respectively. All postoperative refractive error values were statistically significantly different from preoperative values (*p*-value < 0.001).

The *p*-values for the comparison of CDVA and UDVA between preoperative and postoperative visits are shown in Table 2. The mean efficacy index (mean postoperative UDVA/mean preoperative CDVA) was 0.97 ± 0.11 and 0.97 ± 0.19 in the low-myopia group, and 1.14 ± 0.14 and 1.15 ± 0.17 in the high-myopia group at 1 month and 1 year postoperatively, respectively. The safety index (mean postoperative CDVA/mean preoperative CDVA) was 1.09 ± 0.17 and 1.13 ± 0.17 in the low-myopia group, and 1.16 ± 0.13 and 1.22 ± 0.19 in the high-myopia group at 1 month and 1 year, respectively, after surgery.

**Figure 1:**
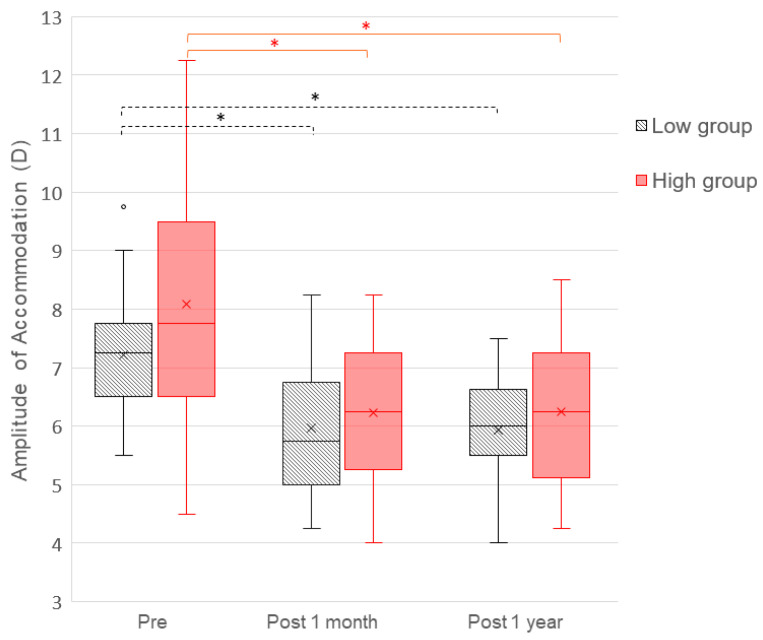
Amplitude of accommodation in diopters (D) over time before and after ICL implantation for two groups: low-myopia (gray-striped boxplot) and high-myopia (red non-striped boxplot). Both graphs show significant differences between the preoperative and the postoperative measurements at 1 month and 1 year (*p*-value < 0.001). There were no significant differences in AA between the groups for any of the follow-up visits. X, mean value; * Statistically significant.

Figure 1 shows the longitudinal change in the amplitude of accommodation (AA) over the 1 year follow-up period. Preoperatively, there were no differences in the AA between the low- and high-myopia groups (*p* = 0.107). Postoperative analyses revealed a statistically significant decrease in the AA in both groups relative to baseline at 1 month and 1 year (*p* < 0.001). There were no statistically significant changes in the AA between 1 month and 1 year postoperatively in either group. In the low-myopia group, the AA decreased from 7.21 ± 1.13 D at baseline to 5.96 ± 1.08 D and 5.93 ± 0.92 D at 1 month and 1 year after surgery, respectively. In the high-myopia group, it decreased from 8.09 ± 2.01 D preoperatively to 6.24 ± 1.23 D and 6.24 ± 1.36 D at 1 month and 1 year postoperatively. There were no statistically significant differences between groups for any of the postoperative AAs at 1 month or 1 year (*p* = 0.467 and 0.445, respectively). Data are shown in Table 3.

The outcomes of the accommodative lag are summarized in Table 3. There were no significant differences in the accommodative lag between the low- and high-myopia groups at any of the follow-up visits (*p*-value > 0.05). Additionally, no statistically significant changes were detected at 1 month or 1 year after surgery compared to the preoperative values in either group (*p*-value > 0.05).

In terms of optical quality, nominal differences were observed in the OSI and SR between the preoperative and 1 year postoperative periods in the low-myopia group (*p* = 0.021 and 0.037, respectively); however, neither value survived Bonferroni correction for multiple comparisons (adjusted α = 0.017), and these should therefore be interpreted as statistical trends rather than confirmed significant findings.

No statistically significant differences were observed during the rest of the follow-up period in either group, nor for any visit comparisons in the high-myopia group. There were no statistically significant differences in the MTF cut-off frequency when postoperative measurements were compared with baseline measurements or with each other in either group (*p*-value > 0.05). Similarly, no statistically significant differences were observed in the preoperative and postoperative follow-up measurements of any of the optical quality parameters when the low- and high-myopia groups were compared (*p*-value > 0.05). Detailed optical quality metrics are presented in Table 4.

**Figure 2:**
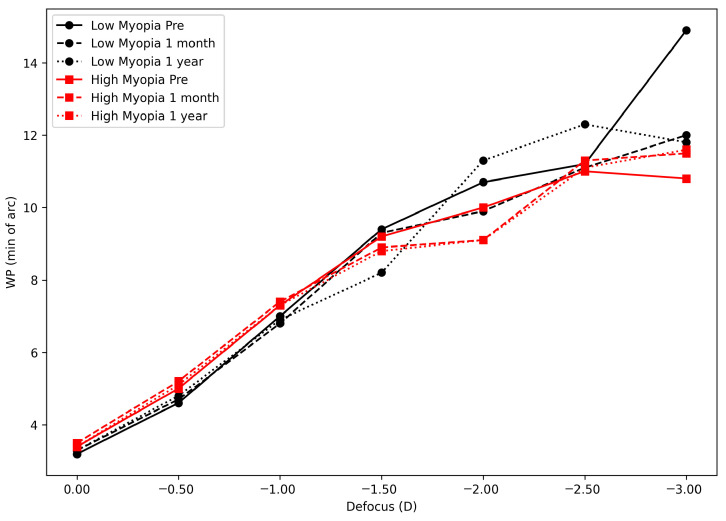
Changes in the objective accommodative response over time before and after ICL implantation in two groups: low-myopia (black line) and high-myopia (red line). The *y*-axis represents the width of the profile at 50% (WP) in minutes of arc. The *x*-axis shows the values for each defocus step tested.

Figure 2 and Table 4 show the width of profile at 50% for each defocus lens point or accommodative demand tested. No statistically significant changes were observed for any of the defocus points between the preoperative and postoperative visits for either group, except for the 1.50 D defocus point, where a statistically significant difference in the WP was found between preoperative and 1 month postoperative (*p* = 0.04) in the low-myopia group.

Regarding the comparison between groups, a statistically significant difference was observed at the 1.50 D defocus point (*p* = 0.04) at 1 month postoperatively. There were no statistically significant intergroup differences found for the remaining defocus points or follow-up visits.

Regarding the ICL vault measurements, the mean ICL vault measurements under mydriatic conditions decreased significantly over the 1 year follow-up period in both groups (Table 5). In the low-myopia group, the vault decreased from 0.41 ± 0.19 mm at 1 month to 0.36 ± 0.17 mm at 1 year (*p* < 0.001). Similarly, in the high-myopia group, the vault decreased from 0.43 ± 0.14 mm to 0.38 ± 0.15 mm at the same time points (*p* < 0.001). No statistically significant differences in the vault were observed between groups at any follow-up visit (*p*-value > 0.05). Vault measurements under miotic conditions showed a similar pattern of reduction over time in both groups (low myopia: *p*-value = 0.009; high myopia: *p* < 0.001), with no intergroup differences detected.

When analyzing the relationship between the ICL vault and accommodative amplitude changes, simple Pearson correlations between the vault and ΔAA were weak and non-significant at both 1 month (r = 0.084, *p* = 0.617) and 1 year (r = 0.088, *p* = 0.621), indicating no direct unadjusted association. However, when preoperative AA was included as a covariate, strong negative partial correlations emerged at 1 month (partial r = −0.757, *p* < 0.001) and 1 year (partial r = −0.747, *p* < 0.001), suggesting that among patients with equivalent baseline accommodation, a higher vault was associated with smaller reductions in the AA. This pattern reflects a suppression effect: patients with a higher preoperative AA show larger absolute reductions regardless of the vault, masking any vault-dependent contribution in the unadjusted analysis. These findings indicate that the relationship between the vault and accommodative changes is not independent of preoperative accommodative reserve, and vault magnitude alone should not be considered a primary determinant of postoperative AA reduction (Table 6).

## 4. Discussion

The present study provides new evidence on the 1 year effects of EVO Visian ICL implantation on accommodative function and optical quality related to accommodation among patients with low and high myopia.

Firstly, after 1 year of follow-up, the implanted lens demonstrated high efficacy and safety, with comparable outcomes between the high- and low-myopia groups. These findings are consistent with previous reports showing stable refractive predictability, minimal residual error, and no loss of corrected visual acuity after ICL implantation [4,21]. The mean efficacy and safety indices at 1 year exceeded 0.95 and 1.10, respectively, comparable to those reported by Pinto et al. [4] and Dougherty and Priver [21]. These results confirm the medium-term visual stability and safety of the procedure.

At 1 year following EVO ICL implantation, the AA remained significantly lower than the baseline values in both myopia groups. The AA showed a mean reduction of 1.3 D in the low-myopia group and a mean decrease of 1.9 D in the high-myopia group. These differences were already evident at the first postoperative month and remained stable throughout follow-up, suggesting that the accommodative change occurs early after surgery and does not recover over time, without evidence of progressive or incremental decline. No significant differences were observed between the 1 month and 1 year follow-up visits, confirming the stability of this reduction over time. Although statistically significant, the reduction remained within normal age-related limits; however, it may be of greater clinical relevance in patients closer to the presbyopic age range. It was also below the repeatability threshold of the minus lens test [22,23], indicating no clinical relevance. This interpretation is further supported by quantitative comparison with published repeatability data for the minus lens technique, which reports test–retest variability of approximately 1.0–1.5 D [22,23]. The mean reductions observed in the present study, 1.3 D in the low-myopia group and 1.9 D in the high-myopia group, fall within or only marginally exceed this range, meaning they cannot be confidently distinguished from inherent measurement variability.

To date, only Kamiya et al. [16] have reported AA outcomes at 1 year after ICL implantation. Their results differed from ours, as they observed a transient decrease in the AA at 1, 3, and 6 months postoperatively, with the values returning to baseline after 1 year. In contrast, our study demonstrated a stable reduction in the AA throughout the follow-up period. Several factors may explain this discrepancy. Firstly, there are methodological differences, as Kamiya et al. [16] employed a binocular push-up-based accommodometer—a subjective technique known to overestimate the AA—whereas the minus lens method used in our study yields values that more closely match those obtained through objective accommodative assessment techniques [22,23,24]. Additionally, differences in the age range and degree of preoperative myopia between studies may have further contributed to the observed discrepancies.

The persistence of lower AA values at 1 year may be explained by a mild biomechanical interaction between the ICL haptics and the ciliary body [10,25], possibly limiting ciliary contractility, and/or by a sustained increase in accommodative demand after the removal of spectacle-induced minification [8,26]. Together, these mechanisms can account for the early reduction and subsequent 1 year stability of accommodative amplitude observed in the present study.

Beyond 1 year, evidence on accommodative behavior after ICL implantation remains limited, as only Kamiya et al. [16] and the present study have assessed this time point. Most previous investigations have focused on shorter follow-up periods (one to six months), generally reporting an early postoperative decrease in accommodative amplitude, more pronounced in high myopia [9,10,14,18,27,28,29]. Some studies noted partial recovery in low myopia or stability in younger cohorts, but the overall findings indicate that the initial reduction in the AA tends to persist during the early postoperative months.

In summary, while short- and medium-term studies have suggested that the reduction in the AA following ICL implantation reflects an early adaptive response rather than a true dysfunction, the present study provides the first robust confirmation of this pattern at 1 year postoperatively. Our findings extend previous evidence by demonstrating that the AA remains stable over time, with no progressive decline or clinically relevant impairment. These results strengthen the view that EVO ICL implantation induces only a transient, physiological adjustment of the accommodative system and does not compromise accommodative performance in this age group or refractive range.

To further understand this adaptation, the accommodative lag was also analyzed, as it provides complementary information about the balance between accommodative stimulus and response. In the present study, the accommodative lag remained stable after EVO ICL implantation, with no statistically significant differences compared to the preoperative values in either myopia group. This finding aligns with previous mid-term reports [10,14,18,29], which also observed preserved accommodative response behavior after ICL implantation. However, the present study extends this evidence to 1 year follow-up, demonstrating that the accommodative response remains stable over time and continues to adjust appropriately to visual stimuli. It should be noted that the accommodative lag may also be influenced by changes in retinal image size following surgery, particularly due to differences between spectacle and intraocular correction. The absence of changes in the accommodative lag further reinforces that, although the postoperative reduction in accommodative amplitude reaches statistical significance, it holds no clinical relevance and does not reflect any deterioration in accommodative performance.

Regarding optical quality, the 1 year results demonstrated a high degree of stability in all objective parameters (OSI, MTF cut-off, and Strehl ratio). After applying Bonferroni correction for multiple comparisons (adjusted α = 0.017), no statistically significant differences between preoperative and postoperative values were confirmed in either group. In the low-myopia group, nominal *p*-values for the OSI and Strehl ratio fell below the uncorrected threshold but did not survive correction, and should be interpreted as statistical trends only.

To our knowledge, there is no longitudinal ICL study specifically tracking optical quality evolution up to 1 year. Aruma et al. [30] and Jiang et al. [31] reported 1 year but without preoperative comparison, so change over time cannot be inferred. Thus, our data provide novel longitudinal evidence on the 1 year stability of double-pass metrics after EVO ICL implantation.

Importantly, although the low-myopia group exhibited a statistically significant increase in the OSI and a decrease in the Strehl ratio at the 1 year follow-up, the magnitude of these changes remained within the normal range expected for healthy eyes, indicating no clinically meaningful deterioration in optical quality. Both groups showed postoperative OSI values close to 1.0 at 1 year, consistent with transparent optical media [32]. No significant preoperative differences in optical quality parameters were observed between groups, confirming comparable baseline conditions.

The slight changes detected in the low-myopia group likely reflect the higher baseline homogeneity and particularly favorable optical quality in this subgroup, which makes double-pass metrics more sensitive to minimal variations in forward scatter, even in the absence of clinically perceptible lens alterations. This phenomenon has been previously described, as the OSI and Strehl ratio are known to respond to subtle fluctuations in media transparency, particularly in younger eyes with clear ocular structures [32,33,34]. In contrast, the high-myopia group exhibited greater baseline variability, so equally small postoperative fluctuations did not reach statistical significance.

Moreover, the absence of significant differences in the MTF cut-off frequency at 1 year—both within and between groups—further supports that these minor variations do not represent the true degradation of retinal image quality. Collectively, these findings suggest that the apparent postoperative changes in double-pass parameters are primarily influenced by baseline variability rather than by any genuine clinical decline in optical performance, although subtle biomechanical modulation cannot be entirely excluded.

The persistent, mild and non-clinical reduction in the AA could indicate a physiological adjustment of the accommodative system rather than a loss of ciliary muscle function or impairment. This is consistent with the negligible changes observed in the OSI and MTF. This supports the idea that the EVO ICL is largely optically and biomechanically neutral.

Previous short-term studies have consistently reported stable optical quality parameters after ICL implantation, in line with our results [1,13,14,35]. Yu et al. [35] and Miao et al. [1] observed similar stability at 1–3 months in eyes with slightly higher myopia, while Kamiya et al. [13] found comparable outcomes in lower myopia. Extending these findings, our 1 year results confirm that optical quality remains preserved over time, reinforcing the medium-term stability of the EVO ICL procedure across different myopic ranges.

Similarly, the objective accommodative response assessed with the double-pass technique showed no significant changes over time, reinforcing the preservation of accommodative performance and optical quality in both refractive groups. Likewise, no intergroup differences were observed, confirming that 1 year accommodative function after EVO ICL implantation is maintained regardless of preoperative myopia level, at the 1 year follow-up. To date, no studies have evaluated the 1 year evolution of this parameter after ICL implantation. However, our findings are consistent with previous short-term studies [14,19,36], which also reported a stable width of the profile (WP) and no alteration in optical behavior during accommodation after surgery and in healthy adults.

The double-pass technique offers an objective and reproducible method for evaluating the accommodative response, overcoming many of the limitations inherent to subjective measurements such as distometry, illumination variability, or residual refractive error [19,36,37,38]. This objective approach is particularly valuable when assessing accommodation, a process that is inherently dynamic and influenced by multiple neural and optical factors. By applying this technique, the present study provides robust evidence of the accommodative neutrality of the EVO Visian ICL over a 1 year period, demonstrating that the physiological accommodative mechanism remains functionally stable over time in both low- and high-myopia patients. These findings are consistent with the minimal biomechanical interference expected from a posterior chamber lens, reinforcing the 1 year safety and optical compatibility of the EVO ICL design.

Although this study was designed to compare two refractive ranges, further stratification within each myopia group could provide a more detailed understanding of how refractive magnitude influences accommodative and optical outcomes after ICL implantation. The principal limitation lies in the potential influence of distometry during the preoperative amplitude of accommodation assessment, as measurements obtained over spectacles, rather than contact lenses, may introduce slight systematic deviations related to vertex distance. Additionally, subjective accommodation measurements are inherently dependent on patient response and testing conditions, which may add variability despite standardized protocols. Future research should not only include broader segmentation by myopic severity and age range, but also compare accommodative behavior after myopia correction with other refractive surgical techniques, to clarify whether the observed accommodative changes are specific to posterior chamber p-IOLs or represent a more general post-refractive adaptation process.

An important finding of this study is the absence of a significant correlation between the ICL vault and the postoperative decrease in accommodative amplitude, challenging the hypothesis that lower vault values might mechanically restrict anterior lens displacement during accommodation. Although the vault decreased significantly from 0.44 mm to 0.36 mm over 1 year, likely reflecting natural ICL settling and lens growth [39], this reduction did not correlate with AA changes.

Even the lowest vault values observed (>0.28 mm) exceeded the physiological lens displacement during accommodation in young adults [40], maintaining sufficient clearance and ruling out mechanical restriction as a primary cause of AA reduction. When controlling for preoperative AA, partial correlations became significant, suggesting that patients with a higher baseline AA experience proportionally greater reductions regardless of the vault, possibly reflecting accommodative reserve or measurement ceiling effects rather than vault-dependent biomechanical restriction. These findings reinforce that the mechanisms underlying AA reduction (haptic–ciliary interaction and altered accommodative demand, as discussed above) are independent of the central vault magnitude. Within the age range studied (mean 32 years) and with normal crystalline lens elasticity, the vault does not appear to limit accommodative performance, though older patients approaching presbyopia may theoretically respond differently.

The apparent discrepancy between the simple Pearson and partial correlation analyses in Table 6 requires careful interpretation. The near-zero unadjusted correlations at 1 month (r = 0.084) and 1 year (r = 0.088) confirm that the vault alone does not predict postoperative AA reduction. However, the strong partial correlations after controlling for preoperative AA (r = −0.757 and −0.747, respectively; *p* < 0.001) indicate that, once baseline accommodative reserve is accounted for, patients with a higher vault tend to experience smaller reductions in the AA. This constitutes a classic suppression effect: preoperative AA is positively correlated both with vault tolerance and with the absolute magnitude of AA reduction, thereby masking any vault-related contribution in the unadjusted analysis. The partial r^2^ of approximately 0.56 at 1 year indicates that the vault explains a substantial proportion of the variance in ΔAA after adjustment, a finding that warrants attention in future studies with larger samples. Nevertheless, given that even the lowest observed vault exceeded the physiological range of crystalline lens displacement during accommodation in young adults, the mechanical restriction of accommodation by the ICL remains an unlikely primary mechanism. These results should therefore be interpreted with caution, as the partial correlation is not independent of preoperative accommodative reserve and may reflect ceiling effects rather than true biomechanical interaction.

Although the reduction in the AA reached statistical significance, its magnitude (1.3 D in low myopia and 1.9 D in high myopia) falls within or close to the reported test–retest variability in the minus lens technique (approximately 1.0–1.5 D) [22,23]. When interpreted in the context of the stable lag, unchanged objective accommodative response, preserved optical quality, and stable vault, this statistically significant finding does not appear to represent a clinically meaningful loss of accommodative function. Furthermore, this reduction did not show progression over time, supporting the interpretation of a stable adaptive recalibration rather than early presbyopic dysfunction. 

Finally, as the study population consisted of young adults with a mean age below 35 years and accommodative ability that remained stable over time, these results should be interpreted with caution when extrapolated to older patients approaching presbyopia, individuals with early-onset presbyopia and residual accommodation, and younger patients presenting with preoperative accommodative dysfunction. In addition, this study was limited to the evaluation of a single phakic intraocular lens model (EVO Visian ICL, STAAR Surgical), and therefore the results may not be directly generalizable to other p-IOL designs with different geometrical and biomechanical characteristics. Therefore, it would be of interest to extend this research to older patients, particularly those aged 40 years and above, to better determine the age at which surgical options for myopia correction in the context of early presbyopia can be considered. However, this is not a straightforward question, as the onset of presbyopia often shifts clinical decision-making toward alternative strategies rather than ICL implantation. In this age range, approaches such as micro-monovision or extended-depth-of-focus (EDOF) intraocular lenses may be more appropriate, provided that the residual accommodative capacity can remain stable over time.

## 5. Conclusions

Our 1 year study demonstrates that accommodative function remains stable 1 year after ICL implantation in healthy patients with low and high myopia, as supported by both objective and subjective assessment methods. Although a postoperative decrease in accommodation amplitude was observed, this variation occurred early after surgery and remained stable over time, and was not clinically relevant in our relatively young study population. Similarly, the optical quality parameters, including the MTF cut-off frequency, Strehl ratio and OSI, remained consistent over time for both degrees of myopia. These findings indicate that ICL implantation preserves accommodative performance and optical quality in the medium term, regardless of myopia level. This supports the safety and visual stability of the procedure. These results are clinically relevant because they confirm that ICL implantation does not compromise accommodative function, even in high myopes who represent a growing surgical demographic. Nevertheless, further research involving more diverse populations, including patients with a broader age range, different refractive errors, and pre-existing or reduced accommodative function, is needed to confirm the clinical significance of the current findings.

## Figures and Tables

**Table 1:** Preoperative demographics and baseline data (mean ± SD and range). Comparison between the low- and high-myopia groups (*p*-value).

	Total Group (N = 38)	Low-Myopia Group (N = 19)	High-Myopia Group (N = 19)	*p*-Value
Male/female, N (%)	15 (39.5%)/23 (60.5%)	10 (52.6%)/9 (47.4%)	13 (68.4%)/6 (31.6%)	
Age (y)	31.97 ± 5.36 (20 to 40)	33.11 ± 4.99 (20 to 40)	30.84 ± 5.63 (22 to 40)	0.146
Pre manifest sphere (D)	−6.64 ± 2.42 (−12.25 to −2.50)	−4.87 ± 1.06 (−6.25 to −2.50)	−8.41 ± 2.07 (−12.25 to −6.50)	<0.001 *
Pre manifest cylinder (D)	−0.46 ± 0.38 (−1.25 to 0.00)	−0.63 ± 0.37 (−1.25 to 0.00)	−0.29 ± 0.33 (−0.75 to 0.00)	0.006 *
Pre SE (D)	−6.85 ± 2.39 (−12.25 to −2.88)	−5.15 ± 1.14 (−6.62 to −2.88)	−8.55 ± 2.10 (−12.25 to −6.50)	<0.001 *
ICL power (D)	−7.93 ± 2.37 (−13.00 to −3.50)	−6.21 ± 1.16 (−8.00 to −3.50)	−9.66 ± 1.97 (−13.00 to −7.50)	<0.001 *
Calculated SE target (D)	0.05 ± 0.17 (−0.33 to 0.35)	0.05 ± 0.17 (−0.33 to 0.35)	0.06 ± 0.18 (−0.22 to 0.29)	0.516
Photopic pupil (mm)	3.39 ± 0.63 (2.32 to 4.77)	3.43 ± 0.61 (2.32 to 4.48)	3.35 ± 0.67 (2.33 to 4.77)	0.194
Mesopic pupil (mm)	5.99 ± 1.22 (3.10 to 8.20)	6.09 ± 0.96 (4.7 to 7.7)	5.9 ± 1.43 (3.1 to 8.2)	0.874

Pre, preoperative; SE, spherical equivalent; y, years; D, diopter; SD, standard deviation; mm, millimeters; ICL, implantable collamer lens. * Statistically significant.

**Table 2:** Comparison of UDVA and CDVA over time in low- and high-myopia groups: baseline, 1 month and 1 year postoperative (mean ± SD and *p*-value).

	Follow-Up	Low-Myopia Group (N = 19)	High-Myopia Group (N = 19)	*p*-Value Between Groups
UDVA (logMAR)	Pre	1.06 ± 0.19	1.82 ± 0.76	<0.001 *
	1 month post	−0.06 ± 0.05	−0.08 ± 0.05	0.246
	1 year post	−0.05 ± 0.06	−0.09 ± 0.06	0.062
	*p*-value between visit	Pre vs. 1 month: <0.001 * Pre vs. 1 year: <0.001 * 1 month vs. 1 year: 0.632	Pre vs. 1 month: <0.001 * Pre vs. 1 year: <0.001 * 1 month vs. 1 year: 0.480	
CDVA (logMAR)	Pre	−0.07 ± 0.04	−0.03 ± 0.04	0.014 *
	1 month post	−0.09 ± 0.04	−0.09 ± 0.04	0.773
	1 year post	−0.12 ± 0.06	−0.12 ± 0.06	0.760
	*p*-value between visit	Pre vs. 1 month: 0.190 Pre vs. 1 year: <0.001 * 1 month vs. 1 year: 0.079	Pre vs. 1 month: <0.001 * Pre vs. 1 year: <0.001 * 1 month vs. 1 year: 0.027 *	

SD, standard deviation; logMAR, logarithm of the minimum angle of resolution; CDVA, corrected distance visual acuity; UDVA, uncorrected distance visual acuity; Pre, preoperative; Post, postoperative. * Statistically significant.

**Table 3:** 1 year accommodative changes following ICL implantation in low- and high-myopia groups: baseline, 1 month and 1 year postoperative (mean ± SD and *p*-value).

	Follow-Up	Low-Myopia Group (N = 19)	High-Myopia Group (N = 19)	*p*-Value Between Groups
AA (D)	Pre	7.21 ± 1.13	8.09 ± 2.01	0.107
	1 month post	5.96 ± 1.08	6.24 ± 1.23	0.467
	1 year post	5.93 ± 0.92	6.24 ± 1.36	0.445
	*p*-value between visit	Pre vs. 1 month: <0.001 * Pre vs. 1 year: <0.001 * 1 month vs. 1 year: 0.764	Pre vs. 1 month: <0.001 * Pre vs. 1 year: <0.001 * 1 month vs. 1 year: 0.914	
Acc lag (D)	Pre	0.16 ± 0.49	0.01 ± 0.48	0.325
	1 month post	0.14 ± 0.46	0.09 ± 0.37	0.544
	1 year post	0.15 ± 0.40	0.24 ± 0.47	0.474
	*p*-value between visit	Pre vs. 1 month: 0.968 Pre vs. 1 year: 0.824 1 month vs. 1 year: 0.426	Pre vs. 1 month: 0.277 Pre vs. 1 year: 0.114 1 month vs. 1 year: 0.176	

SD, standard deviation; Pre, preoperative; Post, postoperative; AA, amplitude of accommodation; Acc lag, accommodation lag; D, diopter. * Statistically significant.

**Table 4:** Comparison of optical quality metrics and objective accommodative parameters based on them for low- and high-myopia groups: baseline, 1 month and 1 year postoperative (mean ± SD and *p*-value).

	Follow-Up	Low-Myopia Group (N = 19)	High-Myopia Group (N = 19)	*p*-Value Between Groups
OSI	Pre	0.87 ± 0.31	1.45 ± 1.48	0.488
	1 month post	0.97 ± 0.38	1.07 ± 0.73	0.795
	1 year post	1.05 ± 0.37	1.16 ± 0.82	0.838
	*p*-value between visit	Pre vs. 1 month: 0.196 Pre vs. 1 year: 0.021 1 month vs. 1 year: 0.39	Pre vs. 1 month: 0.307 Pre vs. 1 year: 0.489 1 month vs. 1 year: 0.36	
MTF cut-off (c/d)	Pre	38.47 ± 8.21	34.20 ± 9.79	0.234
	1 month post	34.58 ± 6.65	38.06 ± 9.23	0.271
	1 year post	34.41 ± 7.90	38.45 ± 10.58	0.193
	*p*-value between visit	Pre vs. 1 month: 0.077 Pre vs. 1 year: 0.102 1 month vs. 1 year: 0.981	Pre vs. 1 month: 0.085 Pre vs. 1 year: 0.113 1 month vs. 1 year: 0.554	
SR	Pre	0.22 ± 0.05	0.19 ± 0.06	0.195
	1 month post	0.20 ± 0.04	0.21 ± 0.05	0.522
	1 year post	0.19 ± 0.05	0.20 ± 0.06	0.588
	*p*-value between visit	Pre vs. 1 month: 0.201 Pre vs. 1 year: 0.037 1 month vs. 1 year: 0.17	Pre vs. 1 month: 0.136 Pre vs. 1 year: 0.79 1 month vs. 1 year: 0.635	
WP (min of arc) at defocus of:				
0.00 D	Pre	3.16 ± 0.76	3.52 ± 1.6	0.708
	1 month post	3.25 ± 0.89	3.41 ± 1.31	0.84
	1 year post	3.30 ± 0.89	3.29 ± 0.57	0.413
	*p*-value between visit	Pre vs. 1 month: 0.763 Pre vs. 1 year: 0.356 1 month vs. 1 year: 0.619	Pre vs. 1 month: 0.647 Pre vs. 1 year: 0.586 1 month vs. 1 year: 0.636	
0.50 D	Pre	4.59 ± 1.49	4.94 ± 1.85	0.544
	1 month post	4.76 ± 1.46	5.24 ± 1.94	0.354
	1 year post	4.78 ± 1.39	5.19 ± 2.08	0.786
	*p*-value between visit	Pre vs. 1 month: 0.778 Pre vs. 1 year: 0.381 1 month vs. 1 year: 0.758	Pre vs. 1 month: 0.557 Pre vs. 1 year: 0.687 1 month vs. 1 year: 0.962	
1.00 D	Pre	7.27 ± 2.07	7.28 ± 2.05	0.75
	1 month post	6.72 ± 2.51	7.29 ± 2.89	0.435
	1 year post	6.87 ± 2.12	7.41 ± 2.93	0.548
	*p*-value between visit	Pre vs. 1 month: 0.198 Pre vs. 1 year: 0.565 1 month vs. 1 year: 0.619	Pre vs. 1 month: 0.514 Pre vs. 1 year: 0.937 1 month vs. 1 year: 0.679	
1.50 D	Pre	9.43 ± 2.28	9.32 ± 3.24	0.885
	1 month post	8.16 ± 4.32	8.82 ± 1.91	0.04 *
	1 year post	9.18 ± 4.02	9.16 ± 3.76	0.786
	*p*-value between visit	Pre vs. 1 month: 0.04 * Pre vs. 1 year: 0.227 1 month vs. 1 year: 0.309	Pre vs. 1 month: 0.433 Pre vs. 1 year: 0.776 1 month vs. 1 year: 0.906	
2.00 D	Pre	10.66 ± 3.83	9.98 ± 3.51	0.954
	1 month post	9.88 ± 6.47	9.25 ± 2.95	0.246
	1 year post	11.37 ± 6.45	9.10 ± 3.47	0.496
	*p*-value between visit	Pre vs. 1 month: 0.084 Pre vs. 1 year: 0.653 1 month vs. 1 year: 0.076	Pre vs. 1 month: 0.157 Pre vs. 1 year: 0.586 1 month vs. 1 year: 0.653	
2.50 D	Pre	11.39 ± 4.78	11.04 ± 4.02	0.773
	1 month post	11.18 ± 7.73	11.09 ± 4.29	0.391
	1 year post	12.33 ± 7.01	11.50 ± 5.61	0.873
	*p*-value between visit	Pre vs. 1 month: 0.845 Pre vs. 1 year: 0.636 1 month vs. 1 year: 0.535	Pre vs. 1 month: 0.528 Pre vs. 1 year: 0.796 1 month vs. 1 year: 0.535	
3.00 D	Pre	14.95 ± 8.23	10.77 ± 5.24	0.150
	1 month post	12.06 ± 9.29	11.60 ± 6.51	1
	1 year post	11.77 ± 7.71	11.59 ± 6.66	0.616
	*p*-value between visit	Pre vs. 1 month: 0.112 Pre vs. 1 year: 0.311 1 month vs. 1 year: 0.182	Pre vs. 1 month: 0.756 Pre vs. 1 year: 0.286 1 month vs. 1 year: 0.477	

SD, standard deviation; D, diopter; OSI, objective scatter index; MTF, modulation transfer function; SR, Strehl ratio; c/d, cycles/degree; min of arc, minutes of arc; WP, width of the profile at 50%. * Statistically significant.

**Table 5:** ICL vault measurements under mydriatic and miotic conditions for low- and high-myopia groups: 1 month and 1 year postoperative and between-group comparison (mean ± SD and *p*-value).

	Follow-Up	Low-Myopia Group	High-Myopia Group	*p*-Value Between Groups
Vault Mydriasis (mm)	1 month post	0.41 ± 0.19	0.43 ± 0.14	0.868
	1 year post	0.36 ± 0.17	0.38 ± 0.15	0.668
	*p*-value between visit	<0.001 *	<0.001 *	—
Vault Miosis (mm)	1 month post	0.31 ± 0.16	0.34 ± 0.14	0.884
	1 year post	0.28 ± 0.14	0.28 ± 0.14	0.494
	*p*-value between visit	0.014 *	<0.001 *	—
Vault Range (mm)	1 month post	0.10 ± 0.06	0.10 ± 0.05	0.304
	1 year post	0.09 ± 0.05	0.09 ± 0.05	0.465
	*p*-value between visit	0.328	0.926	—

Vault range, difference between mydriatic and miotic measurements; mm, millimeters. * Statistically significant.

**Table 6:** Correlation analysis between ICL vault and changes in accommodative amplitude.

**Time Point**	**Pearson r**	***p*** **-Value**	**95% CI**
Vault (1 month) vs. ΔAA (Pre—1 month)	0.084	0.617	[−0.213, 0.355]
Vault (1 year) vs. ΔAA (Pre—1 year)	0.088	0.621	[−0.151, 0.326]
**Partial Correlation (Controlling for Preoperative AA)**			
**Time Point**	**Partial r**	***p*** **-Value**	**95% CI**
Vault (1 month) vs. ΔAA (Pre—1 month)	−0.757	<0.001 *	[−0.882, −0.510]
Vault (1 year) vs. ΔAA (Pre—1 year)	−0.747	<0.001 *	[−0.877, −0.509]

Vault measurements obtained under mydriatic conditions using swept-source anterior segment OCT. Partial correlation analysis controls for preoperative accommodative amplitude as a covariate, revealing the independent relationship between vault and AA changes. No adverse effects were reported during surgery or throughout the postoperative follow-up period. ΔAA, change in amplitude of accommodation (decrease from baseline); CI, confidence interval based on 1000 bootstrap samples. * Statistically significant.

## Data Availability

The data presented in this study are not publicly available due to privacy and ethical restrictions.
